# Bis(3,5,7-triaza-1-azoniatricyclo­[3.3.1.1^3,7^]deca­ne) bis­(1,2-dicyano­ethene-1,2-dithiol­ato)nickelate(II)

**DOI:** 10.1107/S160053681101645X

**Published:** 2011-05-07

**Authors:** Chen Pan, Bin Cai, Wen-Bo Pei

**Affiliations:** a Department of Applied Chemistry, College of Science, Nanjing University of Technology, Nanjing 210009, People’s Republic of China

## Abstract

The asymmetric unit of the title complex, (C_6_H_13_N_4_)_2_[Ni(C_4_N_2_S_2_)_2_], comprises one 1-azonia-3,5,7-triaza­tricyclo­[3.3.1.1^3,7^]decane cation and one half of an [Ni(mnt)_2_]^2−^ (mnt^2−^ is maleonitrile­dithiol­ate or 1,2-dicyano­ethene-1,2-dithiol­ate) dianion. The Ni^2+^ ion is located on a center of inversion and is coordinated by four S atoms from two mnt^2−^ ligands in a square-planar coordination mode. Inter­molecular N—H⋯N hydrogen-bond inter­actions link one anion and two cations in the crystal structure.

## Related literature

For general background to square-planar *M*[dithiol­ene]_2_ complexes acting as magnetic materials or showing non-linear optical properties, see: Duan *et al.* (2010 ▶). For the synthesis, see: Pei *et al.* (2010 ▶). For related structures, see: Ren *et al.* (2002 ▶). For related literature on spectroscopic properties, see: Bigoli *et al.* (2002 ▶).
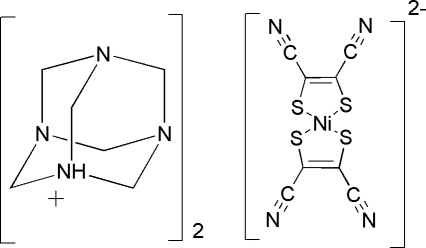


## Experimental

### 

#### Crystal data

(C_6_H_13_N_4_)_2_[Ni(C_4_N_2_S_2_)_2_]
*M* *_r_* = 621.50
Monoclinic, 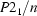

*a* = 10.2274 (9) Å
*b* = 10.7676 (10) Å
*c* = 12.7030 (11) Å
β = 112.212 (2)°
*V* = 1295.1 (2) Å^3^
*Z* = 2
Mo *K*α radiation
μ = 1.11 mm^−1^
*T* = 296 K
0.20 × 0.15 × 0.15 mm

#### Data collection

Siemens SMART CCD area-detector diffractometer
Absorption correction: multi-scan (*SADABS*; Sheldrick 2002 ▶) *T*_min_ = 0.819, *T*_max_ = 0.847
7528 measured reflections
2530 independent reflections
1784 reflections with *I* > 2σ(*I*)
*R*_int_ = 0.049

#### Refinement

*R*[*F* ^2^ > 2σ(*F* ^2^)] = 0.038
*wR*(*F* ^2^) = 0.075
*S* = 1.00
2530 reflections
173 parameters
H atoms treated by a mixture of independent and constrained refinement
Δρ_max_ = 0.48 e Å^−3^
Δρ_min_ = −0.33 e Å^−3^

### 

Data collection: *SMART* (Siemens, 1996 ▶); cell refinement: *SAINT* (Siemens, 1996 ▶); data reduction: *SAINT*; program(s) used to solve structure: *SHELXS97* (Sheldrick, 2008 ▶); program(s) used to refine structure: *SHELXL97* (Sheldrick, 2008 ▶); molecular graphics: *SHELXTL* (Sheldrick, 2008 ▶); software used to prepare material for publication: *SHELXTL*.

## Supplementary Material

Crystal structure: contains datablocks I, global. DOI: 10.1107/S160053681101645X/im2281sup1.cif

Structure factors: contains datablocks I. DOI: 10.1107/S160053681101645X/im2281Isup2.hkl

Additional supplementary materials:  crystallographic information; 3D view; checkCIF report

## Figures and Tables

**Table 1:** Hydrogen-bond geometry (Å, °)

*D*—H⋯*A*	*D*—H	H⋯*A*	*D*⋯*A*	*D*—H⋯*A*
N6—H1⋯N1^i^	0.87 (4)	2.37 (4)	2.941 (5)	124 (3)

Symmetry code: (i) 
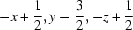
.

**Fig. 1.**
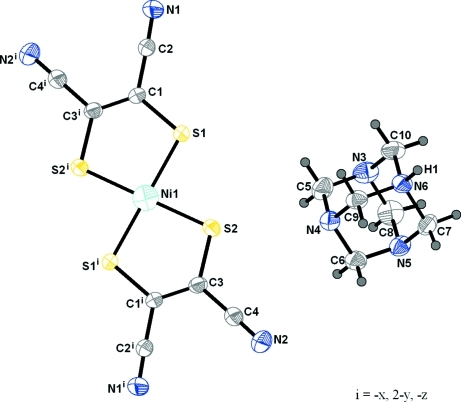
Molecular structure of (I). Displacement ellipsoids are drawn at the 50% probability level (i = -x, 2-y, -z).

**Fig. 2.**
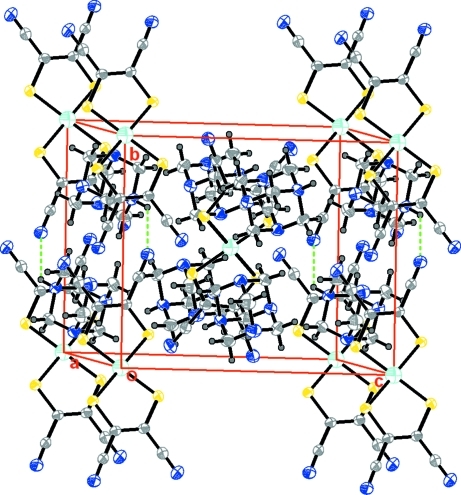
Packing diagram for (I). N—H···N hydrogen bonds are shown as dashed lines.

## supplementary crystallographic information

### Comment

Square-planar *M*[dithiolene]_2_ complexes have been widely studied due to their novel properties and application in the areas of conducting and magnetic materials, dyes, non-linear optics, catalysis and others. These applications arise due to a combination of functional properties, specific geometries and intermolecular interactions (Duan *et al.*, 2010; Pei *et al.*, 2010). Herein we report the crystal structure of the title compound.

The molecular structure of (I) is illustrated in Fig. 1., bond distances and bond angles are given as Supplementary Material. The N—H···N hydrogen bond properties are given in Table 1.

In the asymmetric unit of the title complex, (C_6_H_13_N_4_)_2_(C_8_N_4_NiS_4_), (I), one 1-azonia-3,5,7-triaza-tricyclo[3.3.1.1^3,7^]decane cation and one half of a [Ni(mnt)_2_]^2-^ (mnt^2-^ = maleonitriledithiolate) dianion are observed. The Ni^2+^ ion is located on a crystallographic center of inversion and is coordinated by four S-atoms from two mnt^2-^ ligands in a square-planar coordination mode. Intermolecular N—H···N hydrogen bond interactions join together one anion and two cations of (I) in the crystal structure.

### Experimental

Disodium maleonitriledithiolate (456 mg, 2.5 mmol) and nickel chloride hexahydrate (297 mg, 1.25 mmol) were mixed under stirring in water (20 ml) and heated to boiling about 20 min. After filtering the red solution, an aequeous solution of hexamethylenetetramine hydrochloride (442 mg, 2.5 mmol) was added dropwise to the filtrate. The immediately formed dark red precipitate was filtered off, washed with water and dried in vacuum. The crude product was recrystallized to give red crystals (yield: 645 mg, 83%). Single crystals with block shape suitable for X-ray analysis were obtained *via* recrystallization of the corresponding complex in acetone.

### Refinement

Non-hydrogen atoms were refined anisotropically, whereas the H atom of the NH function was found in a difference Fourier map and was refined isotropically with N—H = 0.86 Å; and the H atoms of methylene protons were calculated and placed to the bonded parent atoms in geometrically idealized positions (C—H = 0.97 Å) and refined as riding atoms, with *U*_iso_(H) = 1.2*U*_eq_(C).

### Crystal data

**Table:**

(C_6_H_13_N_4_)_2_[Ni(C_4_N_2_S_2_)_2_]	*F*(000) = 644
*M**_r_* = 621.50	*D*_x_ = 1.594 Mg m^−^^3^
Monoclinic, *P*2_1_/*n*	Melting point: 448 K
Hall symbol: -P 2yn	Mo *K*α radiation, λ = 0.71073 Å
*a* = 10.2274 (9) Å	Cell parameters from 8518 reflections
*b* = 10.7676 (10) Å	θ = 2.2–26.0°
*c* = 12.7030 (11) Å	µ = 1.11 mm^−^^1^
β = 112.212 (2)°	*T* = 296 K
*V* = 1295.1 (2) Å^3^	Block-shaped, red
*Z* = 2	0.2 × 0.15 × 0.15 mm

### Data collection

**Table:**

Siemens SMART CCD area-detector diffractometer	2530 independent reflections
Radiation source: fine-focus sealed tube	1784 reflections with *I* > 2σ(*I*)
graphite	*R*_int_ = 0.049
φ and ω scans	θ_max_ = 26.0°, θ_min_ = 2.2°
Absorption correction: multi-scan (*SADABS*; Sheldrick 2002)	*h* = −12→12
*T*_min_ = 0.819, *T*_max_ = 0.847	*k* = −13→9
7528 measured reflections	*l* = −15→15

### Refinement

**Table:**

Refinement on *F*^2^	Primary atom site location: structure-invariant direct methods
Least-squares matrix: full	Secondary atom site location: difference Fourier map
*R*[*F*^2^ > 2σ(*F*^2^)] = 0.038	Hydrogen site location: inferred from neighbouring sites
*wR*(*F*^2^) = 0.075	H atoms treated by a mixture of independent and constrained refinement
*S* = 1.00	*w* = 1/[σ^2^(*F*_o_^2^) + (0.0279*P*)^2^] where *P* = (*F*_o_^2^ + 2*F*_c_^2^)/3
2530 reflections	(Δ/σ)_max_ < 0.001
173 parameters	Δρ_max_ = 0.48 e Å^−^^3^
0 restraints	Δρ_min_ = −0.33 e Å^−^^3^

### Special details

**Table:**

Experimental. Anal. Calcd. for C20H26N12NiS4: C, 38.65; H, 4.22; N, 27.05%.Found: C, 38.69; H, 4.19; N, 27.04%. FT—IR data (KBr pellets, cm-1): 3461 (m), 3118 (s), 2202 (s), 1650 (m), 1479 (s), 1257 (s), 1008 (s).
Geometry. All e.s.d.'s (except the e.s.d. in the dihedral angle between two l.s. planes) are estimated using the full covariance matrix. The cell e.s.d.'s are taken into account individually in the estimation of e.s.d.'s in distances, angles and torsion angles; correlations between e.s.d.'s in cell parameters are only used when they are defined by crystal symmetry. An approximate (isotropic) treatment of cell e.s.d.'s is used for estimating e.s.d.'s involving l.s. planes.
Refinement. Refinement of *F*^2^ against ALL reflections. The weighted *R*-factor *wR* and goodness of fit *S* are based on *F*^2^, conventional *R*-factors *R* are based on *F*, with *F* set to zero for negative *F*^2^. The threshold expression of *F*^2^ > σ(*F*^2^) is used only for calculating *R*-factors(gt) *etc*. and is not relevant to the choice of reflections for refinement. *R*-factors based on *F*^2^ are statistically about twice as large as those based on *F*, and *R*- factors based on ALL data will be even larger.

### Fractional atomic coordinates and isotropic or equivalent isotropic displacement parameters (Å^2^)

**Table:**

	*x*	*y*	*z*	*U*_iso_*/*U*_eq_	
C1	0.1231 (3)	1.2643 (3)	0.0413 (2)	0.0280 (7)	
C2	0.1719 (3)	1.3813 (3)	0.0941 (2)	0.0340 (7)	
C3	−0.0743 (3)	0.7460 (3)	0.0733 (2)	0.0293 (7)	
C4	−0.0880 (3)	0.6437 (3)	0.1406 (2)	0.0365 (8)	
C5	0.1017 (3)	0.1801 (3)	0.5935 (2)	0.0482 (9)	
H5A	0.0671	0.2124	0.6494	0.058*	
H5B	0.1362	0.0965	0.6162	0.058*	
C6	0.1637 (3)	0.3842 (3)	0.5569 (2)	0.0416 (8)	
H6A	0.1299	0.4190	0.6124	0.050*	
H6B	0.2399	0.4365	0.5547	0.050*	
C7	0.0982 (3)	0.3365 (3)	0.3613 (2)	0.0408 (8)	
H7A	0.1731	0.3893	0.3573	0.049*	
H7B	0.0222	0.3356	0.2869	0.049*	
C8	−0.0650 (3)	0.3039 (3)	0.4492 (3)	0.0505 (9)	
H8A	−0.1421	0.3029	0.3755	0.061*	
H8B	−0.1007	0.3376	0.5039	0.061*	
C9	0.2719 (3)	0.2090 (3)	0.5110 (2)	0.0380 (8)	
H9A	0.3093	0.1261	0.5334	0.046*	
H9B	0.3478	0.2615	0.5086	0.046*	
C10	0.0343 (3)	0.1257 (3)	0.3993 (3)	0.0461 (9)	
H10A	−0.0421	0.1245	0.3251	0.055*	
H10B	0.0667	0.0412	0.4194	0.055*	
N1	0.2058 (3)	1.4760 (3)	0.1335 (2)	0.0540 (8)	
N2	−0.1037 (3)	0.5627 (3)	0.1924 (2)	0.0576 (9)	
N3	−0.0157 (3)	0.1754 (3)	0.4820 (2)	0.0454 (7)	
N4	0.2179 (3)	0.2575 (2)	0.59227 (18)	0.0382 (6)	
N5	0.0478 (3)	0.3842 (2)	0.44391 (19)	0.0377 (6)	
N6	0.1541 (3)	0.2049 (2)	0.3949 (2)	0.0347 (6)	
Ni1	0.0000	1.0000	0.0000	0.02636 (15)	
S1	0.11966 (8)	1.13766 (7)	0.12444 (5)	0.0364 (2)	
S2	0.00440 (9)	0.88177 (8)	0.14071 (5)	0.0384 (2)	
H1	0.189 (3)	0.175 (3)	0.348 (2)	0.031 (8)*	

### Atomic displacement parameters (Å^2^)

**Table:**

	*U*^11^	*U*^22^	*U*^33^	*U*^12^	*U*^13^	*U*^23^
C1	0.0344 (16)	0.0198 (16)	0.0287 (14)	−0.0032 (14)	0.0108 (12)	0.0003 (12)
C2	0.0470 (19)	0.030 (2)	0.0262 (14)	−0.0073 (16)	0.0150 (14)	0.0020 (14)
C3	0.0359 (17)	0.0241 (17)	0.0312 (14)	−0.0033 (14)	0.0166 (13)	0.0006 (13)
C4	0.0475 (19)	0.033 (2)	0.0295 (15)	−0.0052 (17)	0.0157 (14)	−0.0044 (15)
C5	0.065 (2)	0.043 (2)	0.0456 (18)	−0.0046 (19)	0.0315 (18)	0.0071 (16)
C6	0.056 (2)	0.030 (2)	0.0441 (17)	0.0013 (17)	0.0251 (16)	−0.0042 (15)
C7	0.057 (2)	0.030 (2)	0.0391 (16)	0.0067 (17)	0.0233 (15)	0.0081 (15)
C8	0.042 (2)	0.055 (3)	0.061 (2)	0.0079 (19)	0.0267 (17)	−0.0028 (19)
C9	0.0333 (17)	0.034 (2)	0.0450 (17)	0.0032 (15)	0.0125 (14)	0.0010 (15)
C10	0.049 (2)	0.032 (2)	0.0549 (19)	−0.0128 (17)	0.0175 (16)	−0.0037 (16)
N1	0.084 (2)	0.036 (2)	0.0383 (14)	−0.0203 (17)	0.0189 (14)	−0.0041 (13)
N2	0.094 (2)	0.042 (2)	0.0406 (15)	−0.0115 (18)	0.0292 (16)	0.0059 (15)
N3	0.0440 (16)	0.0391 (17)	0.0607 (17)	−0.0061 (15)	0.0286 (14)	−0.0016 (15)
N4	0.0474 (16)	0.0313 (16)	0.0359 (13)	0.0021 (14)	0.0158 (12)	−0.0007 (12)
N5	0.0460 (15)	0.0306 (17)	0.0420 (14)	0.0071 (13)	0.0228 (12)	0.0028 (12)
N6	0.0437 (16)	0.0305 (17)	0.0354 (13)	0.0040 (13)	0.0212 (12)	−0.0046 (12)
Ni1	0.0352 (3)	0.0193 (3)	0.0253 (2)	−0.0012 (3)	0.0122 (2)	−0.0014 (2)
S1	0.0552 (5)	0.0234 (4)	0.0241 (3)	−0.0058 (4)	0.0076 (3)	0.0014 (3)
S2	0.0607 (5)	0.0292 (5)	0.0253 (4)	−0.0124 (4)	0.0162 (4)	−0.0043 (3)

### Geometric parameters (Å, °)

**Table:**

C1—C3^i^	1.354 (3)	C7—H7B	0.9700
C1—C2	1.425 (4)	C8—N5	1.463 (4)
C1—S1	1.733 (3)	C8—N3	1.477 (4)
C2—N1	1.132 (4)	C8—H8A	0.9700
C3—C1^i^	1.354 (3)	C8—H8B	0.9700
C3—C4	1.433 (4)	C9—N4	1.440 (3)
C3—S2	1.731 (3)	C9—N6	1.512 (3)
C4—N2	1.139 (4)	C9—H9A	0.9700
C5—N4	1.457 (4)	C9—H9B	0.9700
C5—N3	1.471 (4)	C10—N3	1.434 (4)
C5—H5A	0.9700	C10—N6	1.511 (4)
C5—H5B	0.9700	C10—H10A	0.9700
C6—N5	1.477 (3)	C10—H10B	0.9700
C6—N4	1.478 (4)	N6—H1	0.86 (3)
C6—H6A	0.9700	Ni1—S1^i^	2.1751 (7)
C6—H6B	0.9700	Ni1—S1	2.1751 (7)
C7—N5	1.429 (3)	Ni1—S2	2.1810 (7)
C7—N6	1.528 (4)	Ni1—S2^i^	2.1810 (7)
C7—H7A	0.9700		
			
C3^i^—C1—C2	119.9 (3)	N6—C9—H9A	109.8
C3^i^—C1—S1	120.4 (2)	N4—C9—H9B	109.8
C2—C1—S1	119.57 (19)	N6—C9—H9B	109.8
N1—C2—C1	177.4 (4)	H9A—C9—H9B	108.3
C1^i^—C3—C4	120.0 (3)	N3—C10—N6	109.6 (3)
C1^i^—C3—S2	121.0 (2)	N3—C10—H10A	109.7
C4—C3—S2	119.06 (19)	N6—C10—H10A	109.7
N2—C4—C3	177.7 (3)	N3—C10—H10B	109.7
N4—C5—N3	112.5 (2)	N6—C10—H10B	109.7
N4—C5—H5A	109.1	H10A—C10—H10B	108.2
N3—C5—H5A	109.1	C10—N3—C5	109.3 (3)
N4—C5—H5B	109.1	C10—N3—C8	108.7 (3)
N3—C5—H5B	109.1	C5—N3—C8	107.8 (3)
H5A—C5—H5B	107.8	C9—N4—C5	109.6 (2)
N5—C6—N4	111.5 (2)	C9—N4—C6	108.6 (2)
N5—C6—H6A	109.3	C5—N4—C6	108.5 (2)
N4—C6—H6A	109.3	C7—N5—C8	109.4 (3)
N5—C6—H6B	109.3	C7—N5—C6	109.5 (2)
N4—C6—H6B	109.3	C8—N5—C6	108.2 (2)
H6A—C6—H6B	108.0	C10—N6—C9	109.8 (2)
N5—C7—N6	109.2 (2)	C10—N6—C7	108.1 (2)
N5—C7—H7A	109.8	C9—N6—C7	108.7 (2)
N6—C7—H7A	109.8	C10—N6—H1	111.8 (19)
N5—C7—H7B	109.8	C9—N6—H1	107.4 (17)
N6—C7—H7B	109.8	C7—N6—H1	111 (2)
H7A—C7—H7B	108.3	S1^i^—Ni1—S1	180.0
N5—C8—N3	112.1 (2)	S1^i^—Ni1—S2	91.69 (3)
N5—C8—H8A	109.2	S1—Ni1—S2	88.31 (3)
N3—C8—H8A	109.2	S1^i^—Ni1—S2^i^	88.31 (3)
N5—C8—H8B	109.2	S1—Ni1—S2^i^	91.69 (3)
N3—C8—H8B	109.2	S2—Ni1—S2^i^	180.00 (4)
H8A—C8—H8B	107.9	C1—S1—Ni1	103.24 (9)
N4—C9—N6	109.3 (2)	C3—S2—Ni1	103.06 (9)
N4—C9—H9A	109.8		

Symmetry codes: (i) −*x*, −*y*+2, −*z*.

### Hydrogen-bond geometry (Å, °)

**Table:**

*D*—H···*A*	*D*—H	H···*A*	*D*···*A*	*D*—H···*A*
N6—H1···N1^ii^	0.87 (4)	2.37 (4)	2.941 (5)	124 (3)

Symmetry codes: (ii) −*x*+1/2, *y*−3/2, −*z*+1/2.
